# Quality and Reliability of Liver Cancer–Related Short Chinese Videos on TikTok and Bilibili: Cross-Sectional Content Analysis Study

**DOI:** 10.2196/47210

**Published:** 2023-07-05

**Authors:** Shusen Zheng, Xinyu Tong, Dalong Wan, Chen Hu, Qing Hu, Qinghong Ke

**Affiliations:** 1 Department of Hepatobiliary and Pancreatic Surgery The First Affiliated Hospital Zhejiang University School of Medicine Hangzhou China

**Keywords:** liver cancer, short videos, information quality, social media, TikTok, Bilibili, GQS, global quality score, DISCERN, reliability

## Abstract

**Background:**

Liver cancer incidence has been increasing in China in the recent years, leading to increased public concern regarding the burden of this disease. Short videos on liver cancer are disseminated through TikTok and Bilibili apps, which have gained popularity in recent years as an easily accessible source of health information. However, the credibility, quality, and usefulness of the information in these short videos and the professional knowledge of the individuals uploading health information–based videos in these platforms have not yet been evaluated.

**Objective:**

Our study aims to assess the quality of the information in Chinese short videos on liver cancer shared on the TikTok and Bilibili short video–sharing platforms.

**Methods:**

In March 2023, we assessed the top 100 Chinese short videos on liver cancer in TikTok and Bilibili (200 videos in total) for their information quality and reliability by using 2 rating tools, namely, global quality score (GQS) and the DISCERN instrument. Correlation and Poisson regression analyses were applied to discuss the factors that could impact video quality.

**Results:**

Compared to Bilibili, TikTok is more popular, although the length of the videos on TikTok is shorter than that of the videos on Bilibili (*P*<.001). The quality of the short videos on liver cancer in TikTok and Bilibili was not satisfactory, with median GQS of 3 (IQR 2-4) and 2 (IQR 1-5) and median DISCERN scores of 5 (IQR 4-6) and 4 (IQR 2-7), respectively. In general, the quality of videos sourced from professional institutions and individuals was better than that of those sourced from nonprofessionals, and videos involving disease-related knowledge were of better quality than those covering news and reports. No significant differences were found in the quality of videos uploaded by individuals from different professions, with the exception of those uploaded by traditional Chinese medicine professionals, which demonstrated poorer quality. Only video shares were positively correlated with the GQS (*r*=0.17, *P*=.01), and no video variables could predict the video quality.

**Conclusions:**

Our study shows that the quality of short videos on health information related to liver cancer is poor on Bilibili and TikTok, but videos uploaded by health care professionals can be considered reliable in terms of comprehensiveness and content quality. Thus, short videos providing medical information on TikTok and Bilibili must be carefully considered for scientific soundness by active information seekers before they make decisions on their health care management.

## Introduction

Liver cancer is currently the fourth most common malignant tumor and the second leading cause of cancer-related deaths in China, thereby posing a serious threat to people’s lives and health [1]. The main causes of liver cancer include hepatitis B infection, hepatitis C infection, alcohol consumption, nonalcoholic steatohepatitis, and aflatoxin and aristolochic acid exposure [2,3]. Approximately 84% of liver cancers in China are caused by hepatitis B virus infection [4]. Early antiviral treatment plays an extremely important role in delaying the progression of cirrhosis to liver cancer [2]. In recent years, the incidence of liver cancer caused by obesity and alcohol intake has increased, but changing potential patients’ lifestyles through health education can reduce the liver cancer incidence to a certain extent [5]. Treatment for liver cancer includes surgical resection, interventional therapy, liver transplantation, radiation therapy, and systemic therapy. Early detection, diagnosis, and treatment of liver cancer are critical for improving patient outcomes [3].

With increasing penetrance of internet technology, electronic information has gradually replaced paper-based information, and people tend to use the internet as a tool to obtain health information. A study of 12,970 cancer survivors in the United States revealed that patients who were dissatisfied with health care services were more likely to search for web-based health information [6]. In contrast to traditional textual information, which takes a long time to read, videos that are interesting and more visual are becoming increasingly popular [7]. Short-form video-sharing platforms have become more popular in recent years, resulting in an increase in the number of health-related short videos available to the public. Nevertheless, concerns have been expressed regarding the quality and content of these short videos [8]. Because videos can be freely uploaded in the absence of a filtering system, videos on short-form video-sharing platforms often demonstrate poor content quality and reliability; in fact, some even convey deceptive and misleading content. The possibility of encountering incorrect health information in short videos increases patients’ risks who may make health decisions based on inaccurate information. However, recent studies have shown that social media–based interventions (eg, Kakao Talk–based liver cancer prevention program) can promote hepatitis B virus surveillance and liver cancer prevention [9]. Many researchers have studied the quality of videos on different diseases in traditional video-sharing social media platforms such as YouTube [10-13]; however, the recent short-form video apps have not been sufficiently investigated. TikTok, a representative of the short-form video hosting services, has over 100 million users worldwide in over 150 countries and has been downloaded over 20 billion times in the United States alone [8]. Bilibili has millions of active users per month due to its convenience, interactivity, and diversity. Several studies have shown an increase in health information being disseminated though social media platforms in the past decade [14,15]. Researchers have studied the quality of TikTok and Bilibili videos on more than 30 dermatology-related diseases [16]; over 20 COVID-19–related items [14]; approximately 10 psychiatric-psychological–related items [17]; weight loss [18]; internal diseases such as chronic obstructive pulmonary disease [19]; diabetes [20]; and diseases requiring surgery such as gallstones [8], lung nodules [21], and gastric cancer [22]. Although hepatocellular carcinoma videos on YouTube have been evaluated, liver cancer–related videos on TikTok and other social media platforms are yet to be assessed [23]. Therefore, we surveyed the top 100 videos related to liver cancer on TikTok and Bilibili, recorded the video quality by using GQS, determined the video reliability by using DISCERN scores, and analyzed and illustrated the associations between video quality and video source, content, duration, likes, comments, shares, and saves. Our study aims to evaluate the content, quality, and reliability of short videos on liver cancer in Bilibili and TikTok.

## Methods

### Ethical Considerations

No clinical data, human specimens, or laboratory animals were used in this study. All information was obtained from publicly released TikTok and Bilibili videos, and none of the data involved personal privacy concerns. In addition, this study did not involve any interaction with users; therefore, no ethics review was needed.

### Search Strategy and Data Collection

**Figure 1:**
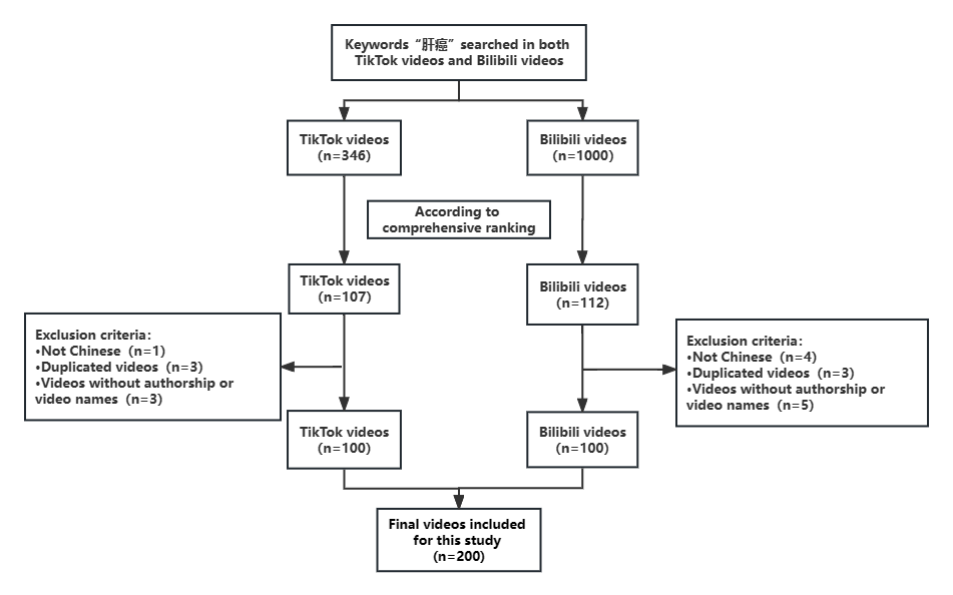
Search strategy for short videos on liver cancer.

In this cross-sectional study, we used “肝癌” (liver cancer) as the keyword to search the top 100 videos in the Chinese versions of TikTok and Bilibili on March 2, 2023 (Figure 1). New accounts were registered and logged in for each video platform to minimize the bias introduced by personal recommendation algorithms. Comprehensive ranking, which is a combination of the video completion rate (the proportion of people who watched more than 5 s), like rate (the proportion of people who liked the video), comment rate (the number of people who left comments on the video), follow rate (the number of people who followed the author), and upload time, recommended the recently uploaded videos and the most popular videos. Non-Chinese videos, duplicated videos (videos with the same content but with different uploaders), and videos without authorship or video names were excluded until the top 100 videos were displayed. We limited the analysis to the top 100 videos because several studies have confirmed that videos beyond the top 100 have no significant impact on the analysis [8,24,25]. The basic information in the included videos was recorded, including the name of the video; the name and identity of the uploader; the length of the video; the content delivered; the number of likes, comments, shares, and saves it received; and the number of days since it was published. All extracted data were recorded in Excel (Microsoft Corp).

### Classification of Videos

We divided the videos into 4 groups according to the source and into 5 groups according to the content. Video sources were categorized as follows: (1) professional individuals, (2) nonprofessional individuals, (3) professional institutions, and (4) nonprofessional institutions. The video content was categorized as follows: (1) disease knowledge, (2) treatment, (3) prevention, (4) news and reports, and (5) advertisement and others. Regarding videos from professional individuals, further classification was performed as follows: (1) doctors specializing in liver cancer–related modern medicine, (2) doctors specializing in other areas of modern medicine, (3) doctors of traditional medicine, and (4) other health care professionals. Specific classification criteria are shown in Multimedia Appendix 1.

### Video Quality and Reliability Assessments

The quality of the information in the videos was assessed using GQS, and the reliability was evaluated using DISCERN. The GQS consists of 5 criteria ranging from 1 to 5; a higher score indicates higher quality [26]. We used the first part of the DISCERN questionnaire to illustrate the reliability of a video—the higher the score was, the better was the reliability. The specific scoring details for GQS and DISCERN are shown in Table 1 and Table 2. We divided the GQS and DISCERN scores into 5 levels, as shown in Table 3.

New accounts were registered and logged in for each video platform to minimize the bias introduced by personal recommendation algorithms. All videos were collected and downloaded by 1 person (XT). The order of the videos was disrupted to reduce the rating sorting bias. The videos were then assessed and scored by 2 qualified doctors (CH and QH) who had a long history of performing hepatobiliary and pancreatic surgeries. Before scoring the videos, the 2 raters reviewed the DISCERN and GQS scoring instructions and discussed the details to prevent cognitive biases. The arbitrator (QK) assigned the final score if the 2 experts’ scores were inconsistent. All authors then agreed on all ratings. Furthermore, we used Cohen κ to quantify the agreement between the 2 raters. According to the criteria proposed by Landis and Koch, a value of κ>0.8 represents excellent consistency, a value between 0.6 and 0.8 is considered substantial, a value between 0.4 and 0.6 is considered moderate, and values <0.4 are considered poor [27].

**Table 1:** Description of the global quality score (5-point scale) for evaluating the quality of the videos with liver cancer information.

Scale	Description
1	Poor quality, poor flow of the site, most information missing, not at all useful for patients
2	Generally poor quality and poor flow, some information listed but many important topics missing, of very limited use to patients
3	Moderate quality, suboptimal flow, some important information is adequately discussed but others poorly discussed, somewhat useful for patients
4	Good quality and generally good flow, most of the relevant information is listed, but some topics not covered, useful for patients
5	Excellent quality and excellent flow, very useful for patients

**Table 2:** Description of the DISCERN instrument for evaluating the reliability of the videos with liver cancer information.

DISCERN	Description
Question 1	Are the aims clear?
Question 2	Does it achieve its aims?
Question 3	Is it relevant?
Question 4	Is it clear what sources of information were used to compile the publication (other than the author or producer)?
Question 5	Is it clear when the information used or reported in the publication was produced?
Question 6	Is it balanced and unbiased?
Question 7	Does it provide details of additional sources of support and information?
Question 8	Does it refer to areas of uncertainty?

**Table 3:** The 5-level scores of global quality score and DISCERN.

Scale, score		Level
**Global quality score**		
	1	Very poor
	2	Poor
	3	Fair
	4	Good
	5	Excellent
**DISCERN**		
	1	Unreliable
	2-3	Less reliable
	4-5	Fairly reliable
	6-7	Relatively reliable
	8	Reliable

### Statistical Analyses

Since our data were nonparametrically distributed, the median (IQR) was used for the descriptive statistics. The Kruskal-Wallis test was used to assess the differences between groups, and Dunn multiple comparison test was used for 2-way intergroup comparisons. We used Cohen κ to quantify the agreement between the 2 raters. We performed Spearman correlation analysis to evaluate the relationship between quantitative variables. Poisson regression models were used to assess the effects of video variables on video quality. The *P* value for the significance of the statistical analysis was <.05. GraphPad Prism version 9.0.0 for Windows was used for data analysis.

## Results

### Video Characteristics

We obtained 200 videos for data extraction and analysis based on our keyword search: 100 from TikTok and 100 from Bilibili. The general features of the videos are presented in Table 4, which revealed that TikTok videos had more likes, comments, shares, and saves than Bilibili videos (all *P*<.001), while videos in Bilibili were longer than those in TikTok (*P*<.001). There was no significant difference in the number of days since the videos were published (*P*=.81).

**Table 4:** Characteristics of the videos in TikTok and Bilibili.

Variable	TikTok (n=100), median (IQR)	Bilibili (n=100), median (IQR)	Wilcoxon rank-sum test	
			*z* score	*P* value
Likes	5706 (1012-26,250)	229 (37-2568)	6.41	<.001
Comments	305 (85-1635)	65 (4-286)	5.42	<.001
Shares	425 (152-3621)	37 (6-358)	6.88	<.001
Saves	576 (174-7691)	139 (20-804)	4.83	<.001
Days since published	344 (118-485)	284 (139-619)	0.23	.81
Duration	71 (38-134)	148 (100-232)	5.76	<.001
Global quality score	3 (2-4)	2 (1-5)	1.92	.055
DISCERN score	5 (4-6)	4 (2-7)	1.47	.14

**Figure 2:**
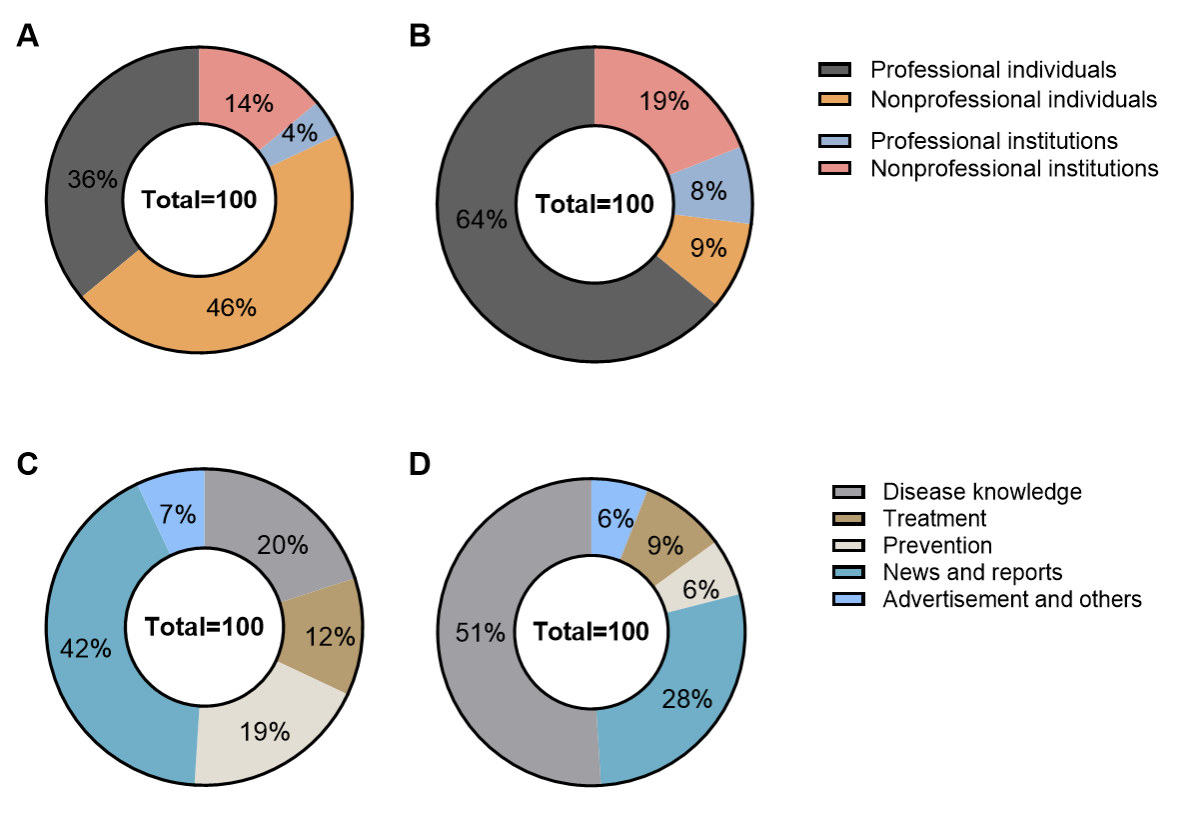
Percentage of videos on liver cancer from different sources and with different contents in TikTok and Bilibili. (A) Sources of Bilibili videos. (B) Sources of TikTok videos. (C) Content types of Bilibili videos. (D) Content types of TikTok videos.

Table 5, Table 6, and Figure 2 show the video source and content type on TikTok and Bilibili. On TikTok, professional individuals uploaded the highest number of videos, accounting for 64% (64/100), followed by nonprofessional institutions (19/100, 19%), nonprofessional individuals (9/100, 9%), and professional institutions (8/100, 8%). Regarding video content on TikTok, most videos conveyed disease knowledge (51/100, 51%), followed by news and reports (28/100, 28%), treatment (9/100, 9%), prevention (6/100, 6%), and advertisement and other information (6/100, 6%). On Bilibili, people preferred videos from nonprofessional individuals (46/100, 46%) and those covering news and reports (42/100, 42%).

**Table 5:** Characteristics of the videos across sources and content in TikTok.

Variable		Likes	Comments	Shares	Saves	Days since published	Duration
**Video sources (n=100), median (IQR)**							
	Professional individuals (n=64)	3120 (888-10,602)	211 (72-709)	367 (142-2301)	339 (143-3215)	310 (85-433)	69 (49-114)
	Nonprofessional individuals (n=9)	48,000 (23,407-188,500)	3281 (265-15,540)	3953 (620-13,500)	11000 (1736-13,500)	295 (66-468)	192 (137-263)
	Professional institutions (n=8)	879 (35-1568)	195 (4-494)	132 (7-191)	163 (30-755)	384 (175-1255)	59 (32-126)
	Nonprofessional institutions (n=19)	20,000 (6508-91,000)	1176 (151-12,000)	1373 (258-5008)	2261 (409-23,000)	644 (318-731)	29 (19-137)
**Video content (n=100), median (IQR)**							
	Disease knowledge (n=51)	1598 (597-8622)	155 (49-473)	290 (73-1312)	240 (126-2612)	335 (118-439)	68 (46-95)
	Treatment (n=9)	1693 (581-4379)	262 (76-449)	236 (116-635)	381 (130-673)	274 (126-761)	51 (27-97)
	Prevention (n=6)	18,500 (9110-150,250)	1864 (289-5299)	8769 (3121-18,000)	116,449 (811-22,500)	132 (72-314)	144 (44-167)
	News and reports (n=28)	20,000 (3142-94,000)	985 (164-7802)	1230 (278-4509)	2213 (296-14,250)	424 (280-640)	86 (25-144)
	Advertisement and other information (n=6)	59,500 (31,203-133,500)	7640 (317-22,500)	9473 (756-21,500)	7218 (982-54,250)	186 (21-410)	171 (90-267)

**Table 6:** Characteristics of the videos across sources and content in Bilibili.

Variable		Likes	Comments	Shares	Saves	Days since published	Duration
**Video sources (n=100), median (IQR)**							
	Professional individuals (n=36)	329 (38-6700)	90 (7-427)	52 (4-420)	125 (20-798)	339 (165-654)	125 (70-169)
	Nonprofessional individuals (n=46)	824 (111-2444)	101 (7-279)	46 (11-316)	284 (44-1015)	242 (136-586)	208 (103-437)
	Professional institutions (n=4)	4 (2-11)	0 (0-0)	3 (2-5)	5 (1-6)	761 (151-965)	119 (115-1245)
	Nonprofessional institutions (n=14)	62 (25-5613)	4 (0-722)	11 (3-532)	45 (10-940)	257 (127-411)	158 (117-524)
**Video content (n=100), median (IQR)**							
	Disease knowledge (n=20)	82 (13-1149)	9 (0-107)	17 (3-231)	50 (9-574)	409 (171-727)	191 (110-642)
	Treatment (n=12)	36 (6-434)	5 (0-68)	48 (1-541)	40 (5-1177)	211 (22-339)	129 (46-512)
	Prevention (n=19)	539 (44-7005)	44 (4-679)	81 (9-1945)	409 (26-2584)	243 (83-620)	145 (118-199)
	News and reports (n=42)	1210 (131-5090)	160 (27-597)	49 (8-171)	216 (39-577)	307 (159-648)	130 (70-130)
	Advertisement and others (n=7)	116 (37-4296)	5 (0-144)	50 (10-291)	121 (26-746)	285 (116-510)	215 (134-796)

### Video Quality and Reliability Assessments

**Figure 3:**
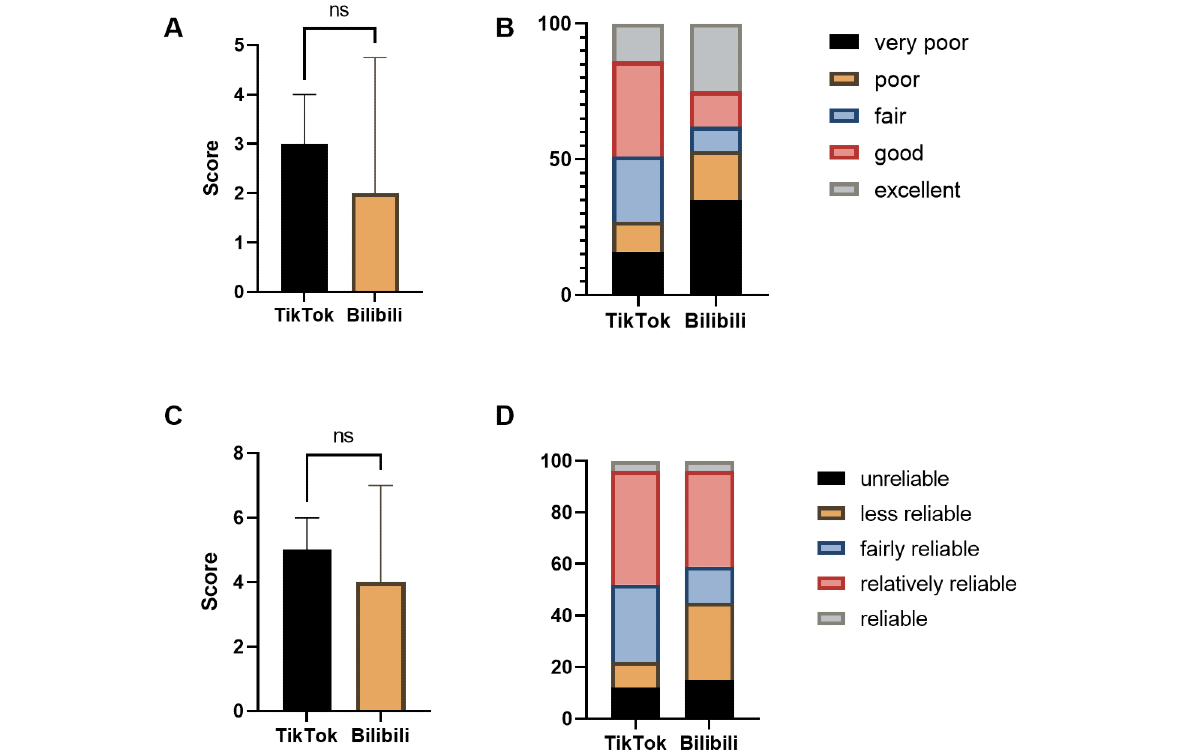
Global quality scores, DISCERN scores, and quality/reliability distributions of short videos related to liver cancer on TikTok and Bilibili. (A) Comparison of global quality scores between TikTok and Bilibili videos. (B) Proportions of different levels of video quality. (C) Comparison of DISCERN scores between TikTok and Bilibili videos. (D) Proportions of different levels of video reliability. ns: not significant at *P*<.05.

The κ value indicating interobserver reliability was 0.75. Table 7 shows the detailed results of the DISCERN and GQS of the videos. Regarding TikTok videos, the GQS median score was 3 (IQR 2-4) and the median DISCERN score was 5 (IQR 4-6), indicating that the TikTok videos were of fair quality and reliability. Regarding Bilibili videos, the GQS median score was 2 (IQR 1-5) and the median DISCERN score was 4 (IQR 2-7), indicating that the Bilibili videos were of poor quality and fair reliability. As shown in Figure 3, no significant difference was observed between the GQS and DISCERN scores of TikTok and Bilibili videos (*P*=.055 and *P*=.14, respectively). However, most Bilibili videos were of poor quality and low reliability.

**Figure 4:**
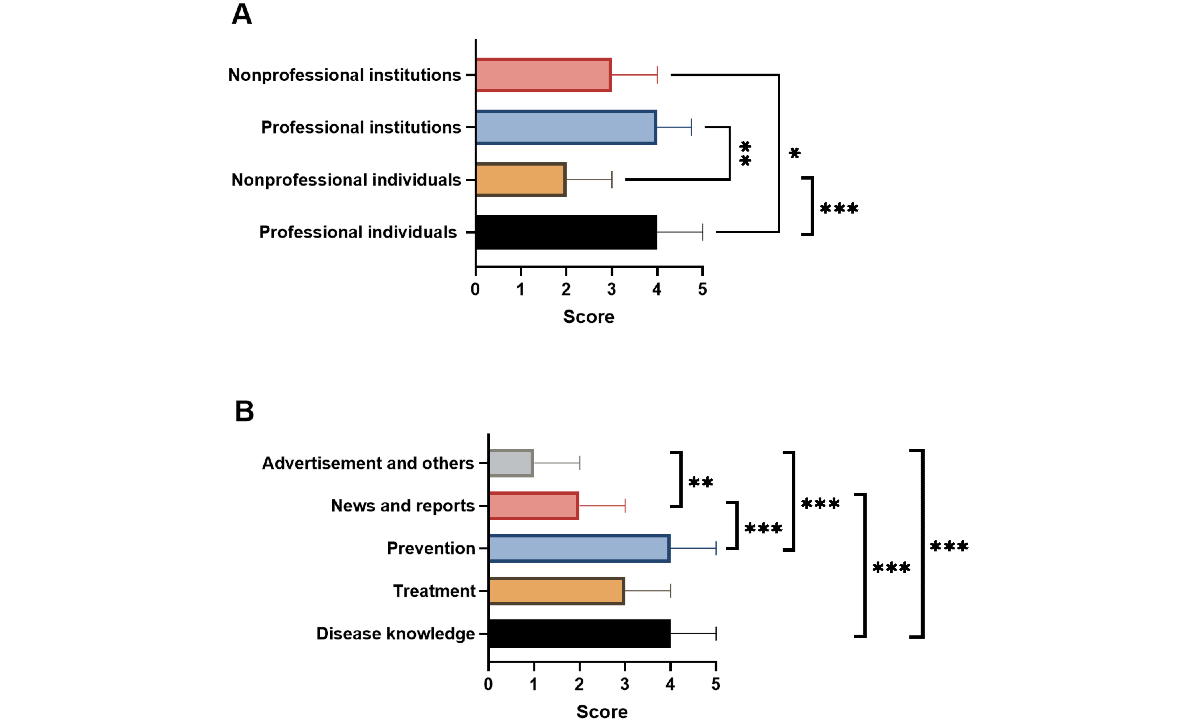
Global quality scores of videos related to liver cancer from different sources (A) and with different contents (B). *P<.05, **P<.01, ***P<.001.

**Figure 5:**
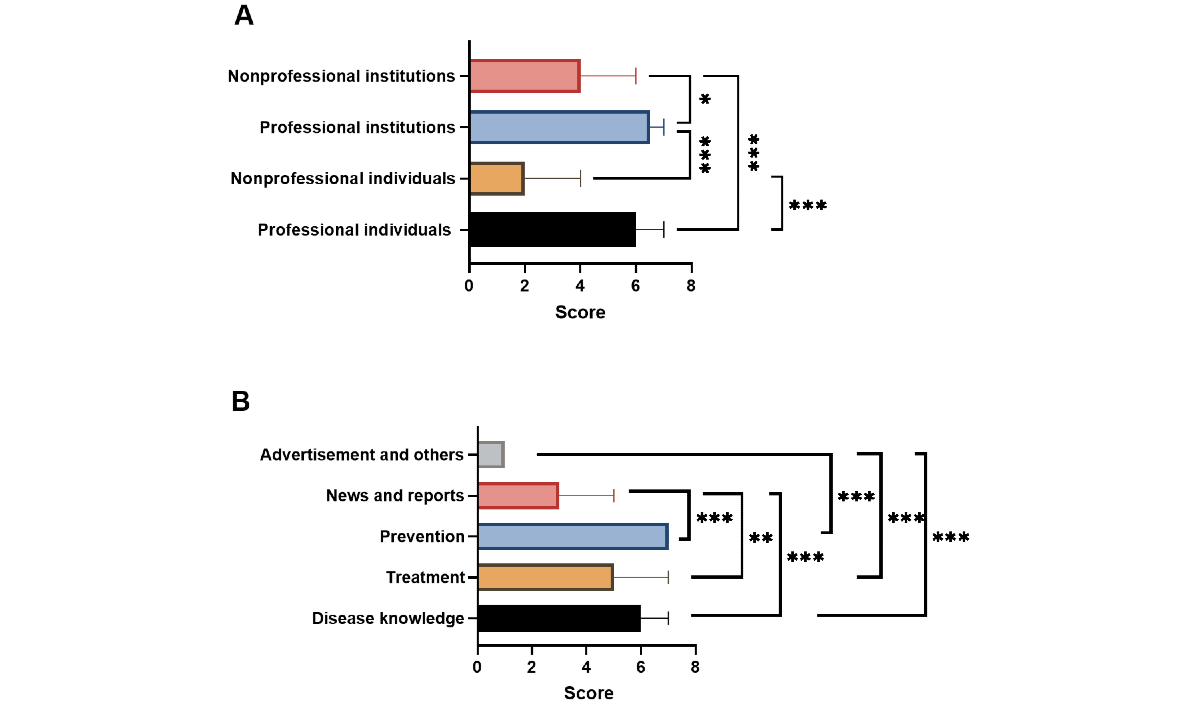
DISCERN scores of videos related to liver cancer from different sources (A) and with different contents (B). *P<.05, **P<.01, ***P<.001.

We compared the GQS and DISCERN scores of videos from different sources and with different contents. Videos by professional individuals and institutes had higher GQS than those by nonprofessional individuals (*P*<.001 and *P*=.002, respectively). Meanwhile, videos covering disease knowledge and prevention had higher GQS than those covering news and reports (*P*<.001 and *P*<.001, respectively) and advertisements and other information (*P*<.001 and *P*<.001, respectively) (Figure 4). Similarly, videos by professional individuals and institutes had higher DISCERN scores than those by nonprofessional individuals (*P*<.001 and *P*<.001, respectively) and institutions (*P*<.001 and *P*<.001, respectively). Meanwhile, videos covering disease knowledge and prevention had higher DISCERN scores than those covering news and reports (*P*<.001 and *P*<.001, respectively) and advertisements and other information (*P*<.001 and *P*<.001, respectively) (Figure 5).

**Figure 6:**
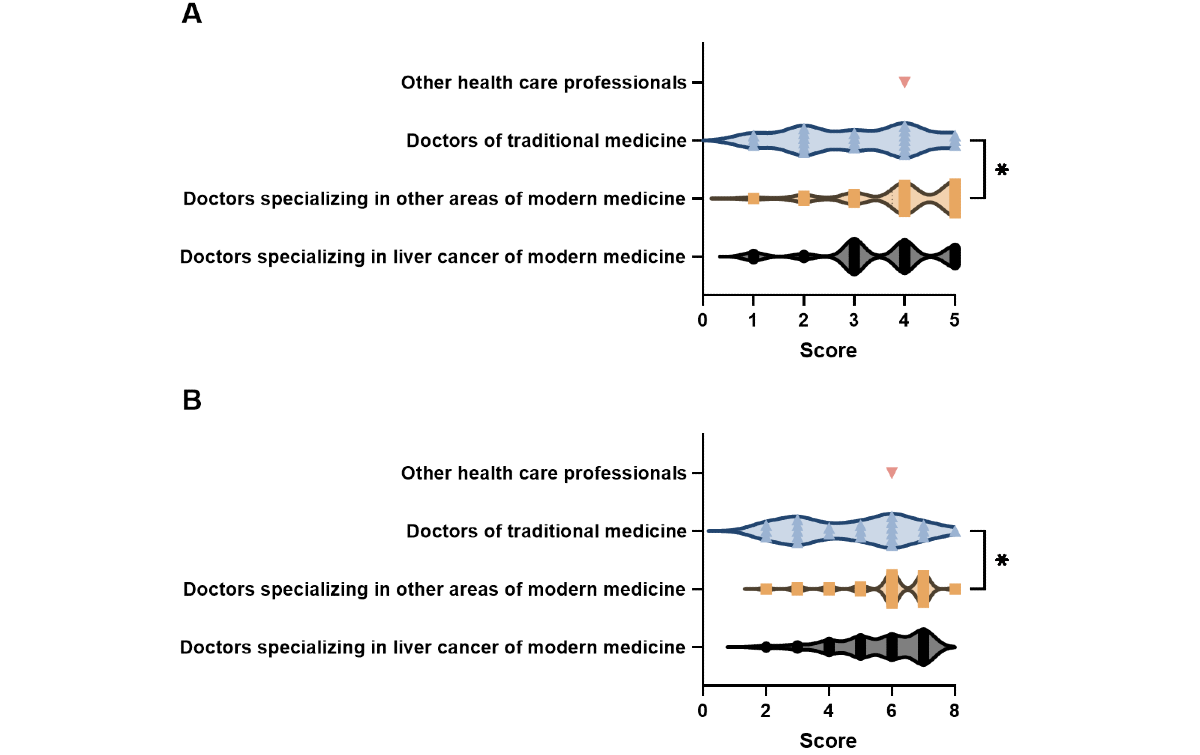
Global quality scores and DISCERN scores of short videos related to liver cancer uploaded by different professional individuals. *P<.05.

To explore whether different types of professional individuals impacted the quality and reliability of the videos, we further divided the professional individual–sourced videos into 4 groups. Videos by doctors of traditional medicine had lower GQS and DISCERN scores than videos by doctors specializing in other areas of modern medicine (*P*=.01 and *P*=.03, respectively). However, no significant differences were found between other source groups (*P*>.99) (Figure 6).

**Table 7:** The 5-level global quality scores and DISCERN scores for TikTok and Bilibili videos related to liver cancer.

Scale, score		TikTok (n=100), n	Bilibili (n=100), n
**Global quality score**			
	1 (very poor)	16	35
	2 (poor)	11	18
	3 (fair)	24	9
	4 (good)	35	13
	5 (excellent)	14	25
**DISCERN**			
	1 (unreliable)	12	15
	2-3 (less reliable)	10	30
	4-5 (fairly reliable)	30	14
	6-7 (relatively reliable)	44	37
	8 (reliable)	4	4

### Correlation Analysis and Poisson Regression Analysis

The data were not normally distributed; thus, we used Spearman correlation analysis to reveal the relationships between different video variables (Table 8). Positive correlations were observed for the following variables: likes and comments (*r*=0.91, *P*<.001), likes and shares (*r*=0.86, *P*<.001), likes and saves (*r*=0.91, *P*<.001), shares and comments (*r*=0.83, *P*<.001), saves and comments (*r*=0.86, *P*<.001), and saves and shares (*r*=0.90, *P*<.001). Meanwhile, the time of video publication and video duration were not significantly related to other variables. Only shares positively correlated with GQS (*r*=0.17, *P*=.01) (Table 9). However, the Poisson regression analysis revealed no significant video variable capable of predicting GQS and DISCERN scores (all *P*>.05; Table 10).

**Table 8:** Spearman correlation analysis between the video variables.

Variable		Likes	Comments	Shares	Saves	Days since published	Duration
**Likes**							
	*r*	1	—^a^	—	—	—	—
	*P* value	—	—	—	—	—	—
**Comments**							
	*r*	0.91	1	—	—	—	—
	*P* value	<.001	—	—	—	—	—
**Shares**							
	*r*	0.86	0.83	1	—	—	—
	*P* value	<.001	<.001	—	—	—	—
**Saves**							
	*r*	0.91	0.86	0.90	1	—	—
	*P* value	<.001	<.001	<.001	—	—	—
**Days since published**							
	*r*	0.08	0.07	0.07	0.08	1	—
	*P* value	.27	.30	.30	.37	—	—
**Duration**							
	*r*	–0.09	–0.003	–0.01	0.04	–0.08	1
	*P* value	.22	.96	.99	.62	.25	—

^a^Not applicable.

**Table 9:** Pearson correlation analysis between video variables and the global quality scores and DISCERN scores.

Variable		Global quality score	DISCERN
**Likes**			
	*r*	–0.03	–1.31
	*P* value	.68	.06
**Comments**			
	*r*	–0.02	–0.11
	*P* value	.81	.12
**Shares**			
	*r*	0.17	0.07
	*P* value	.01^a^	.07
**Saves**			
	*r*	0.03	–0.07
	*P* value	.65	.31
**Days since published**			
	*r*	0.04	0.05
	*P* value	.55	.57
**Duration**			
	*r*	0.03	0.03
	*P* value	.70	.70

^a^Significant at *P*<.05.

**Table 10:** Association between video variables and global quality score and DISCERN score.

Scale, video variable		Relative risk (95% CI)	*P* value
**Global quality score**			
	Likes	1.000000 (0.999999-1.000001)	.75
	Comments	1.000000 (0.999986-1.000015)	.95
	Shares	1.000003 (1.000000-1.000007)	.07
	Saves	1.000001 (1.000000-1.000003)	.12
	Days since published	1.000062 (0.999810-1.000313)	.63
	Duration	0.999876 (0.999588-1.000164)	.40
**DISCERN**			
	Likes	1.000000 (0.999999-1.000000)	.24
	Comments	0.999989 (0.999976-1.000003)	.12
	Shares	1.000002 (0.999999-1.000005)	.26
	Saves	1.000000 (0.999999-1.000002)	.62
	Days since published	1.000037 (0.999834-1.000241)	.72
	Duration	0.999831 (0.999591-1.000072)	.17

## Discussion

### Principal Findings

In this cross-sectional study, we reviewed videos on liver cancer from 2 popular Chinese short-form video-hosting platforms, namely, TikTok and Bilibili. TikTok is more popular, while Bilibili videos are longer. We used GQS and DISCERN scores to evaluate the quality and the reliability of the information uploaded in the videos, respectively. The overall quality of TikTok and Bilibili short videos related to liver cancer was unsatisfactory according to the GQS and DISCERN scores, which may be attributed to the relatively low standards set for access to these platforms and the lack of video censorship. Although no significant differences were observed in the video quality between the 2 platforms (GQS, *P*=.055; DISCERN, *P*=.14), Bilibili seems to have lower quality videos than TikTok, which may result from the higher number of videos uploaded by nonprofessional individuals rather than by professional individuals. Additionally, Bilibili videos covered mostly news and reports as opposed to conveying disease knowledge directly. Videos by professional individuals or institutions and videos wherein the content is primarily concerned with disease knowledge are of higher quality and are more reliable. Except for doctors of traditional medicine whose videos were of poor quality, individuals from other professional backgrounds uploaded higher-quality videos.

### Quality of the Short Videos on Liver Cancer

First, due to the nature of the short video format, the short duration and single presentation mode limits the breadth and richness of the content. People tend to demand more content rather than more in-depth understanding. In particular, in the case of educational videos, a short duration does not always allow for a clear explanation of a disease. Although we did not find a relationship between video length and video quality, this may be related to the fact that we did not compare videos with the same content.

Second, we found that the quality of videos uploaded by professionals and professional institutions was higher than that of videos uploaded by nonprofessionals and institutions, indicating that the subject matter expertise is critical for health care information videos. Given the high demand for professionalism in medical science videos, we believe that short video platforms should introduce thresholds for uploaders of this type of medical science videos. When we collected the videos, both in TikTok and Bilibili, some medical professional uploaders were professionally certified by the platform with special symbols, and the real identity of these uploaders could be confirmed on the official websites of their hospitals, demonstrating that the certification of professionals by TikTok and Bilibili is reliable, thereby eliminating fake information to a certain extent. Professional certification is a tool that other short-video platforms should adopt for increasing the reliability and trustworthiness of health care information videos. Unfortunately, these platforms do not impose complete restrictions on uploaders, and there are still many nonprofessionals uploading medical science videos. For example, our study shows that the proportion of nonprofessional individuals uploading videos on Bilibili is high; such actions may lead to a decline in the quality of medical science videos on short-video platforms.

Third, the audience for these short videos is not professional medical workers, and the time limitation for these videos requires doctors to explain the content more concisely and understandably, which, in part, leads to simpler and shallower content. People also prefer lower-quality news reports or biographies over videos covering medical knowledge, which can be perceived as boring; thus, doctors must use a combination of case studies and knowledge to spread scientific information in a shorter time to grab the reader’s attention and express information in a more interesting way.

### Correlation Between Video Quality and Video Characteristics

We found no significant or only a partial positive correlation between video quality and viewers’ likes, comments, shares, and saves, which was surprisingly contrary to the findings of some previous studies [8,25]. Some studies have shown that TikTok viewers cannot distinguish between high-quality and low-quality videos, while the video popularity indicator negatively correlates with the DISCERN score [8]. This finding is also consistent with previous research on YouTube video quality [25]. Those researchers contributed these circumstances to TikTok’s recommendation mechanism, which dictates that videos with more likes are more likely to be recommended [25]; thus, lower-quality popular videos become more popular, further exacerbating the gap between video quality and popularity. However, in our video search, not only videos with a high number of likes were recommended at the top of the list, some of the latest videos and some of the doctors’ videos were also pushed to the top. Thus, people can choose between 2 different video recommendation models, perhaps with a comprehensive ranking leading to higher-quality videos rather than more popular videos. We also observed a positive correlation between the shares and the quality of the videos, suggesting an improvement in people’s ability to recognize high-quality videos.

### Evaluation of Quantitative Scoring Tools

In this study, we used the GQS and DISCERN scores to evaluate the quality and reliability of the short videos, respectively. The GQS is a 5-point Likert scale that evaluates web-based information by the accuracy, usability, and flow of information [28,29]. Since 1998, DISCERN has been one of the most widely used tools for assessing the quality of health information and has been extensively and successfully used to rate health-related videos on YouTube, Twitter, and Facebook [30]. DISCERN involves a short questionnaire that enables users to assess the quality of health information covering treatment options [31]. DISCERN has worked well in previous studies assessing the quality of health-related video information [19]. The consistency of DISCERN and GQS is acceptable; however, for short videos with less comprehensive content, it is difficult to perform an objective assessment according to the criteria. For example, videos dealing with treatment rarely introduce and explain other aspects of the disease. The Patient Education Materials Assessment tool was used for the first time in a quality assessment of videos on thyroid cancer and was designed to assess the structure, sequence, visual cues, text, illustrations, suggestions, and other aspects of the audiovisual material directly related to the quality of the video [32]. Our study did not use the Patient Education Materials Assessment scoring tool since we were primarily concerned with the quality of textual content; however, it is a useful recommendation for improving the assessment of video content in the future.

### Practical Significance

With the growing interest in internet-based health promotion through improvements in internet technology and people’s need for higher health standards, the internet has changed the patient’s role from a passive recipient of information to an active seeker [20]. With the successful development of multimedia technology and the proliferation of electronic devices such as mobile phones, visual social media is becoming an important source of information for patients. Various social media platforms have enabled convenient access to medical information. However, the uneven quality of the videos poses many problems; some videos deceive consumers and provide incorrect information. Professionals are aware of these dangers, and the Chinese government has recently issued guidelines for the publication and dissemination of scientific health–related knowledge through various media platforms, which is the first guideline for health promotion in the world [33]. Undoubtedly, higher-quality videos should be in the spotlight and receive more attention. A good health-promotion video should combine scientific soundness, popularity, and ease of understanding, and should lack inaccurate and misleading information. Therefore, video quality must be assessed to provide viewers with access to trustworthy information, and future research should provide advice on how these platforms can be built and developed.

### Strengths and Limitations

The strengths of our study are as follows. Our study targets 2 of China’s largest short-video platforms, with TikTok covering viewers of all ages and cultural levels and Bilibili mainly serving a relatively young age group. Assessing these 2 video platforms allowed for more realistic and reliable findings and prevented the limitations associated with using a single research platform. For video assessment, we chose GQS to assess the quality of the videos and DISCERN to assess the reliability of the videos, thereby analyzing the video information from multiple dimensions. This is also the first study in China to analyze the quality of short videos in the field of liver cancer on 2 social media platforms.

However, there are some limitations in our study. First, we only included 100 videos out of the 346 videos on liver cancer in TikTok and 100 videos out of the 1000 videos on liver cancer in Bilibili. Although we included a relatively small percentage of videos, we considered it to be sufficiently representative, as videos beyond the top 100 have no significant impact on the analysis [8,24,25]. Second, we only included videos uploaded on Chinese video-sharing platforms; thus, the findings may not be generalizable to other language platforms. Subsequent cross-linguistic research is required to fill this gap. Third, the assessment tools we chose are more subjective and less comparable across different studies. Although 3 independent experts determined the ratings iteratively and used Cohen κ to quantify the agreement between the 2 raters, subjective differences still cannot be ignored. Additionally, the 2 scoring tools we chose were mainly for published text, and the assessment of video quality was incomplete; for example, it was not possible to evaluate the audio and graphic content of the videos, which calls for more applicable scoring criteria to be proposed. In addition, the small sample size of some content groupings (eg, the professional institutions group) may have introduced inaccuracies. This can be addressed by increasing the sample size or by adding search terms.

### Conclusion

In our study, we collected and assessed the information quality of 200 videos related to liver cancer on 2 main short-form video-sharing social media platforms in China (TikTok and Bilibili). We found that the quality and reliability of the videos on these platforms were unsatisfactory across sources and content. Overall, videos by medical professionals were more instructive in terms of the comprehensiveness, quality, and reliability of content than videos by nonmedical professionals. Videos by professionals conveying knowledge such as disease knowledge are likely to be of higher quality. Thus, given the increasing popularity of video-sharing platforms, it is important to increase the regulation and quality control of such platforms, and people should be careful when accessing information for health care management on short-video platforms.

## Appendix

Multimedia Appendix 1 Classification of videos.

## Abbreviations

GQS: global quality score
