# Supplementary material for: Quality and Reliability of Liver Cancer–Related Short Chinese Videos on TikTok and Bilibili: Cross-Sectional Content Analysis Study
Source: J Med Internet Res. 2023 Jul 5;25:e47210. doi: 10.2196/47210 (PMC10357314; doi:10.2196/47210)
Supplement: Multimedia Appendix 1 [file jmir_v25i1e47210_app1.docx]

| Table S1 Classification of Videos | |
| --- | --- |
| Video Source | |
| 1. Professional Individuals | Individuals who have real name recognition and professional accreditation in medical area, including doctors, nurses and other healthcare professionals. |
| 1. None-professional Individuals | Individuals who do not have real name recognition or professional accreditation in medical area. |
| 1. Professional Institutions | Institutions who have professional accreditation in medical area, including hospitals, medical schools, medical centers and other healthcare institutions. |
| 1. None-professional Institutions | Institutions who do not have professional accreditation in medical area, including newspaper, TV station, network media and other none-professional groups. |
| Source of Professional Individuals | |
| 1. Doctors specializing in Liver Cancer of Modern Medicine | Including specialists in surgical and internal hepatology, surgical oncology, and other doctors who work on liver cancer-related issues. |
| 1. Doctors specializing in other areas of Modern Medicine | Including specialists in other surgery, internal medicine, emergency medicine, imaging, interventional medicine and other areas of medicine |
| 1. Doctors of Traditional Medicine | Doctors specializing in Chinese traditional medicine. |
| 1. Other Healthcare Professionals | Including nurses, epidemiologists, technologists, basic research specialists and other healthcare professionals |
| Video Content | |
| 1. Disease knowledge | Including knowledge of anatomy, epidemiology, basic research, etc. |
| 1. Treatment | Treatment options for liver cancer |
| 1. Prevention | Preventative measures for liver cancer |
| 1. News and Reports | News reports or biographies of liver cancer patients |
| 1. Advertisement and Others | Content with a commercial purpose or irrelevant content which do not involve information of liver cancer |
